# Obstinate Prolapse of the Uterus

**Published:** 1901-10-12

**Authors:** 


### OBSTINATE PROLAPSE OF THE UTERUS.

Many operations have been performed by various gynæcologists for the relief of the misery caused by persistent prolapse of the uterus. In some cases of prolapse all that is required is a comfortably-fitting pessary. If this fail then a plastic operation may be done; the uterus may be fastened to the anterior abdominal wall, or the vagina may be reduced in calibre, or the perineum may be sewn up, or combinations of these various measures may be undertaken, often with very satisfactory results. In some patients, however, so great is the tendency to relaxation of the parts that even scar tissue stretches, and whatever is done to prop up the uterus it persists in coming down again. In such cases the uterus has been removed. The danger of a simple hysterectomy is so small and the misery of a constantly protruding uterus is so great that such an operation is quite justifiable when it succeeds. Unfortunately the very laxity which let down the uterus tends to let down the rest of the pelvic contents, a rectocele and a cystocele appearing where the uterus formerly protruded. Mr. Christopher Martin[1] has, however, devised an operation which, by its thoroughness, deserves the success which it attained in the case in which he performed it. He extirpated not merely the uterus but the whole of the vaginal canal. The uterus, ovaries and tubes, together with the whole of the vaginal mucous membrane were removed in one piece, then the fascia at one side of the vagina was stitched to that at the other side by a fine continuous suture, beginning at the level of the broad ligaments and working downwards to just above the vulva, so pushing the anterior wall of the rectum back and the posterior wall of the bladder forwards by a firm column of connective tissue derived from the lateral fasciæ. The operation was done over two years ago, and the patient remains perfectly comfortable, with no protrusion and with complete control over the bladder. Of course the patient was past the menopause and the operation is only suggested as a *dernier ressort.*

[1] Brit. Med. Jour., Oct. 5.

